# Rapid Automated Target Segmentation and Tracking on 4D Data without Initial Contours

**DOI:** 10.1155/2014/547075

**Published:** 2014-08-03

**Authors:** Venkata V. Chebrolu, Daniel Saenz, Dinesh Tewatia, William A. Sethares, George Cannon, Bhudatt R. Paliwal

**Affiliations:** ^1^Department of Biomedical Engineering, University of Wisconsin-Madison, Madison, WI 53706, USA; ^2^Department of Human Oncology, University of Wisconsin-Madison, Madison, WI 53792, USA; ^3^Wisconsin Institute of Medical Research, 1111 Highland Avenue, Madison, WI 53705, USA; ^4^Department of Medical Physics, University of Wisconsin-Madison, Madison, WI 53792, USA; ^5^Department of Electrical & Computer Engineering, University of Wisconsin-Madison, Madison, WI 53706, USA; ^6^Department of Radiation Oncology, Intermountain Healthcare, Salt Lake City, UT 84107, USA

## Abstract

*Purpose*. To achieve rapid automated delineation of gross target volume (GTV) and to quantify changes in volume/position of the target for radiotherapy planning using four-dimensional (4D) CT. *Methods and Materials*. Novel morphological processing and successive localization (MPSL) algorithms were designed and implemented for achieving autosegmentation. Contours automatically generated using MPSL method were compared with contours generated using state-of-the-art deformable registration methods (using Elastix© and MIMVista software). Metrics such as the Dice similarity coefficient, sensitivity, and positive predictive value (PPV) were analyzed. The target motion tracked using the centroid of the GTV estimated using MPSL method was compared with motion tracked using deformable registration methods. *Results*. MPSL algorithm segmented the GTV in 4DCT images in 27.0 ± 11.1 seconds per phase (512 × 512 resolution) as compared to 142.3 ± 11.3 seconds per phase for deformable registration based methods in 9 cases. Dice coefficients between MPSL generated GTV contours and manual contours (considered as ground-truth) were 0.865 ± 0.037. In comparison, the Dice coefficients between ground-truth and contours generated using deformable registration based methods were 0.909 ± 0.051. *Conclusions*. The MPSL method achieved similar segmentation accuracy as compared to state-of-the-art deformable registration based segmentation methods, but with significant reduction in time required for GTV segmentation.

## 1. Introduction

In the practice of radiation therapy better local control and survival are often associated with increased delivered dose [1]. The greatest limitation to increasing treatment dose is induced by normal lung toxicity. Due to nonperiodic breathing pattern in patients, the planned dose is very often not delivered as intended. Interfractional target motion considerably deteriorates the geometric accuracy of the delivery process. In the recent past, systems and methodologies such as TomoTherapy [2, 3] and cone-beam computer tomography (CBCT) [4, 5] were developed and used in clinical practice to improve treatment planning and delivery. A quick and accurate method of contouring structures would be useful to improve the efficacy of these systems. Manual segmentation is too time-consuming, making rapid imaging and automated target delineation very attractive for motion management in radiation therapy.

A typical four-dimensional (4D) data for radiation treatment planning in lung cancer includes 10 phases (separated by 10% difference from 0 to 100% of the breathing cycle) and approximately 100 images per phase. To estimate target volume and motion, contours for the gross target volume (GTV) are required on all the phases of the 4D data. Manual contouring often used for contouring GTV suffers from being very time consuming and subject to intra- and interobserver variability [6]. Reproducibility of the results with manual contouring is challenging due to variations in the experience and training of radiation oncologists. The problem of segmentation is accentuated by the complexity of tumor geometry and by the relatively similar intensity of the tumors as compared to the surrounding tissues or organs. Importantly, manual segmentation is prone to bias and error and is not suitable for rapid, adaptive treatment planning. The use of autocontouring algorithms is very attractive for introducing dynamic assessment of target shape, volume, and position. This work focuses on the design and implementation of computationally efficient automated image segmentation algorithms for rapid assessment of the shape, volume, and position of the target for radiotherapy planning using 4DCT.

Accurate delineation of the GTV is crucial in treatment planning because the construction of both the clinical target volume (CTV) and the planning target tumor volume (PTV) is based on the GTV. During the radiotherapy process, tumor regression often occurs, and when the change is beyond some threshold, the contours should be adapted to the new GTV. Similarly, accurate delineation of the tumor volume during the different phases of breathing cycle is crucial to reduce margins added around the clinical tumor volume. Further reduction in margin would be anticipated to decrease integral radiation dose, mitigating potential acute and late side effects to organs at risk (OAR), and allowing further dose escalation as indicated. The specific tumor trajectory and position often changes from day to day and during the delivery of radiation therapy treatment. When small margins are used, such variability may be detrimental when the treatment target is in motion. Intrafraction tumor motion due to the respiratory cycle and cardiac motion and interfraction differences in the patient's position, anatomy, tumor size, and shape during the course of treatment often result in suboptimal delivery of the planned radiation dose. Often in practice, an extended volume envelope is used to address the problem of uncertainties due to motion. Unfortunately, this increase in treatment volume limits the patient tolerance dose. Rapid and automatic delineation can therefore improve clinical workflow and efficiency, which can eventually improve the therapeutic ratio.

The state of the art for obtaining CT contours particularly in a 4DCT is deformable registration [7–9] based segmentation. These algorithms use manually created contours on one phase of 4D data to automatically segment the target volume(s) in the other phases of 4D data [10]. Deformable image registration delineates motion of any internal structure as well as deformation from the reference phase to each phase. This registration procedure outputs deformation maps, that is, voxel-to-voxel displacement between the reference image and each phase image. One limitation of deformable registration approach for application in adaptive treatment planning is that, before the deformable registration method can be used to determine target motion profiles, there is a need to manually segment one phase of the 4DCT data. Another limitation of deformable registration is that it is generally computationally complex. In this work we design and implement novel computationally efficient automated segmentation algorithms that do not require manual contouring on one phase of 4DCT data. We apply these autocontouring algorithms to quantify changes in volume/position of the target during free breathing.

## 2. Methods and Materials

Nine lung cancer patients were imaged using 4DCT protocol under free-breathing condition with a GE Discovery LightSpeed CT Scanner (GE Healthcare Waukesha, WI) under the request of a physician interested in target motion. An appropriate institutional review board (IRB) approved the study. The imaging parameters include slice thickness of 2.5 mm, an energy of 120 kVp, and a tube current of 100 mA. Varian Real-time Position Management (RPM) system was used for acquiring the respiratory waveform for retrospective binning. The raw data was retrospectively binned using Advantage 4D software to divide the data into ten breathing phases.

### 2.1. Automated Image Segmentation Using Morphological Processing and Successive Localization

Novel and computationally efficient morphological processing and successive localization (MPSL) algorithms were developed for achieving automated segmentation of the body, lung, and the tumors [11, 12]. Morphological operations such as dilation and erosion are computationally efficient. If *A* and *B* are two subsets in a* N*-dimensional space, then the morphological operation such as dilation and erosion on subset *A* with a structuring element *B* is mathematically represented as

(1) Dilation:A⊕B={c∈ZN ∣ c=a+b  for  some  a∈A,b∈B},Erosion:AΘB={x∈ZN ∣ x+b∈A  for  every  b∈B}.

The following sections describe the utilization of these operations to achieve automated segmentation.

#### 2.1.1. Segmenting the Tumors

Initially, the intensity range for the tumors and the surrounding regions were defined. In general, this a priori information required for automated segmentation can be obtained using the images from a prior CT scan. If there were no overlaps between the intensity range for the tumors and the surrounding regions, then a threshold on the maximum/minimum intensity would segment the tumors. However, in general there will be an overlap between the two intensity ranges. Therefore, a binary mask that includes the regions with intensity values within either of the two intensity ranges was generated.

The binary mask generated above was then eroded to produce disjoint regions. The amount of erosion was determined empirically by performing the erosion operation in a population of individuals. Each of the separate regions in the disjoint region data was then labeled and the volume occupied by each labeled region was determined; that is, after morphologically separating the tumors using erosion, the different regions were labeled (using the union-find algorithm [13]) and then the morphologically connected regions and their volumes were calculated. The labeled regions were analyzed to identify the tumor region. This analysis process identifies regions with volumes close to the range of typical tumor volumes; that is, a limit on the maximum/minimum possible volume of the tumors was used as a filter to isolate the tumors. This filtering based on size removes nontumor regions. This identification can be supplemented with tumor location data if known.

The mask obtained above was dilated by the same amount as the prior erosion to obtain the tumor segmentation; that is, the erosion is reversed through a dilation to restore the tumors to their approximate original size.

The inputs needed for this algorithm are the minimum and maximum intensity threshold values for the tumor and surrounding regions (for generating a mask), the minimum and maximum limits for tumor volume, and the erosion/dilation radius (for morphological processing). The optimal input values for erosion/dilation radii and threshold ranges were empirically chosen. The algorithm was developed to work at both 2 and 3 dimensions.

**Figure 1:**
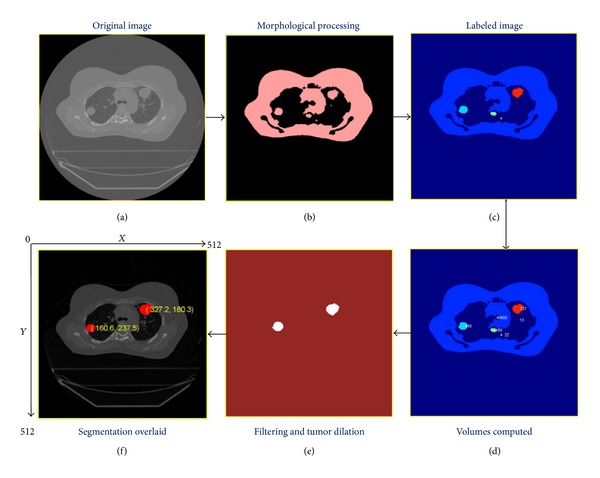
Morphological processing based automated segmentation approach for contouring the tumors. The center of geometry of the segmented tumors is useful for quantifying tumor motion between different phases.

Figure 1 shows the flowchart for the above described segmentation procedure in a specific example. The empirically chosen threshold ranges create a binary image, including the tumor and surrounding tissue with a direct connection between them. Image erosion (shown in Figure 1(b)) removes the connection between the tumor and the lung wall. The result is a postmorphologically processed image with separated tumors. All independent regions (nonconnected volumes) are labeled (Figure 1(c)) and the volume of each disconnected region was calculated (Figure 1(d)). The expected range of tumor volume provided as input allows for localizing the tumor among the labeled regions (Figure 1(e)). Morphological dilation restores tumors to the original size after tumor identification (Figure 1(f)).

#### 2.1.2. Phantom Validation

**Figure 2:**
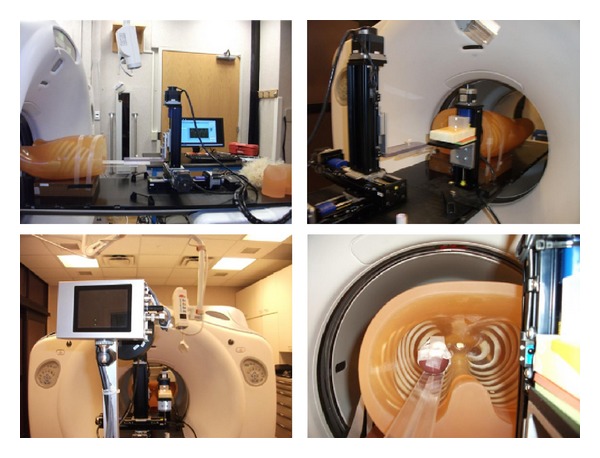
Experimental setup for validating MPSL-based motion and volume quantification using anthropomorphic chest phantom and the Washington University 4D motion system.

The MPSL algorithm was validated using a phantom experiment. The LUNGMAN anthropomorphic chest phantom (Figure 2) was used for the validation. A 6-cm diameter sphere of virtual water was used to simulate the GTV. The Washington University 4D Motion system [14] generated realistic motion profiles, while 4DCT scans were acquired. Figure 2 shows the experimental setup.

### 2.2. Ground-Truth Generation via Manual Contouring

The GTV contours generated by the MPSL algorithm were compared with the contours manually drawn on the different phases of the 4D data, considering the manual contours as “ground-truth.” ITK-SNAP [15] was used to generate manual contours. Manual contours took 1–5 minutes per phase to complete depending on the size of the GTV. The manual contours were generated by one of the investigators (DS). Interobserver variation in generation of manual contours is expected, as shown in other studies [16, 17].

### 2.3. Deformable Registration Based GTV Segmentation

Deformable image registration (DIR) based GTV contours were generated using Elastix© software (a toolbox for deformable image registration) and MIMVista software for comparison with the results from our algorithm. Manual contours were drawn on the first phase of 4DCT data and were then propagated to the other phases using DIR. Elastix© used a b-spline transformation based DIR algorithm with a mutual information similarity metric and rigid penalty described by Staring in 2007 [18]. MIMVista used intensity-based free-form VoxAlign Deformation Engine, previously used in the literature, for propagating manually drawn contours on one phase to the remaining phases in the dynamic study [19, 20].

### 2.4. Motion Estimation

The target volumes as well as the center of geometry (COG) positions were recorded by computing the GTV contour statistics. The COG of the segmented target was used as a measurement of the GTV position, so that GTV trajectory can be estimated. The GTV trajectory (the trajectory of the COG of the GTV) was estimated using MPSL method and was compared with the trajectory generated using MIMVista.

When using MIMVista the GTV in one phase was contoured in a semiautomated manner and the GTV in the remaining phases was segmented using deformable registration. The semiautomated segmentation of the GTV on one phase was performed as follows. A threshold of −83 HU was initially used, allowing voxels with intensity values above that threshold to be included in the contours for the GTV. Next, manual adjustments were made to fill in holes that were erroneously excluded from the GTV and to delete parts of normal anatomy that were included. Then, MIMVista's 4DCT adaptive recontouring tool was used to propagate the contours to the other phases.

### 2.5. Statistics

The comparison between GTV contours generated using MPSL and deformable registration based segmentation were compared with ground-truth using Dice similarity coefficient. Sensitivity, positive-predictive value (PPV) and accuracy in volume quantification were also compared for the contouring methods. The sensitivity measured the fraction of the voxels in the ground-truth that the automatic contour (MPSL/DIR based) included. The PPV measured the fraction of voxels inside the automatic (MPSL/DIR based) contour that were “true positives” (points in the ground-truth contour).

## 3. Results

### 3.1. Accuracy Comparison

In the phantom experiment, the volume and COG motion profiles estimated by the MPSL algorithm were within 5% error of the known ground-truth values.

The accuracy of the contours generated using MPSL and DIR based segmentation in patients was estimated by calculating the Dice similarity coefficient, as well as the sensitivity and PPV in comparison to the ground-truth.

Table 1 shows the results for the nine cases. The two-sided paired *t*-test for statistical difference between the Dice coefficients of MPSL and DIR methods resulted in a *P* value of 0.024. Similarly, the *P* values for statistical difference between MPSL and DIR based methods for Sensitivity and PPV were 0.006 and 0.104, respectively.

**Figure 3:**
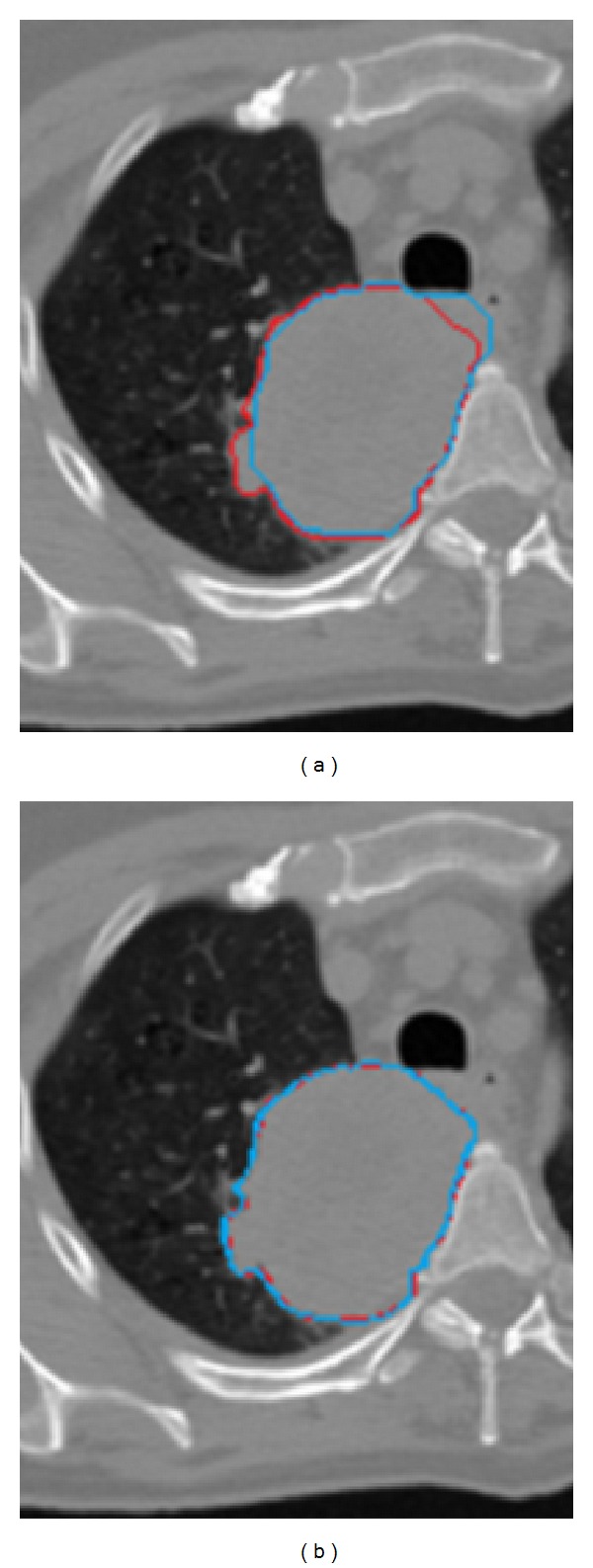
Comparison of automated contouring methods with ground-truth in case 1. (a) Manual contour (red) compared with MPSL (turquoise) based segmentation of GTV. (b) Manual contour (red) compared with DIR based contour (turquoise).

**Figure 4:**
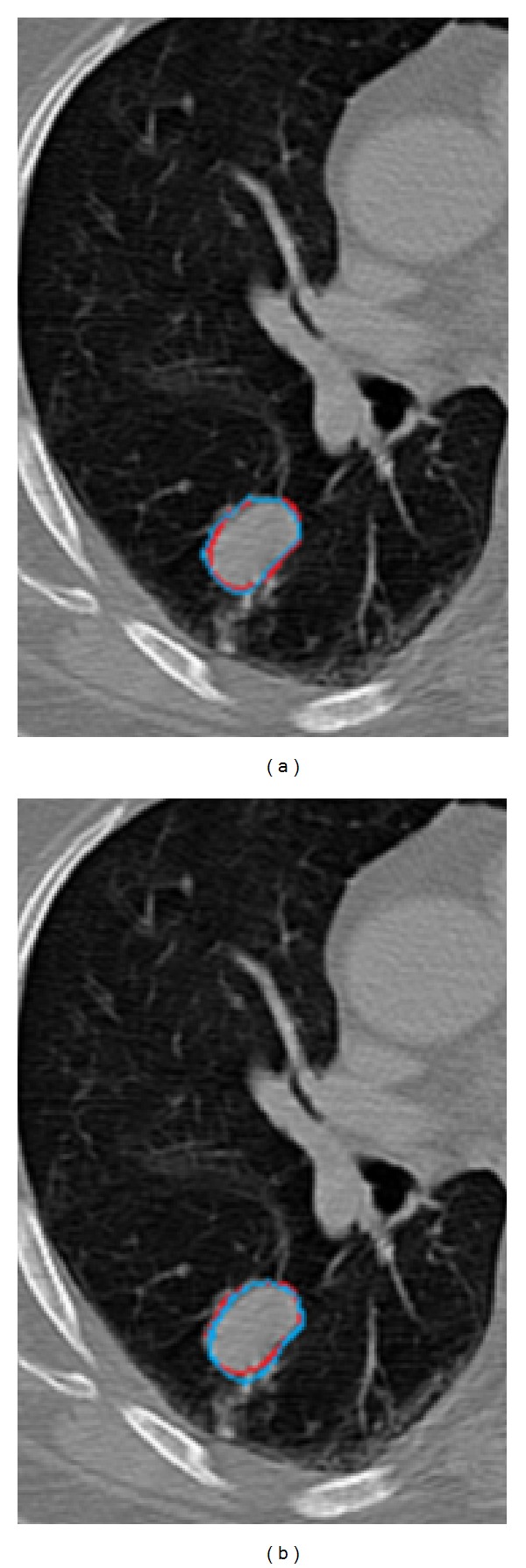
Comparison of automated contouring methods with ground-truth in case 2. (a) Manual contour (red) compared with MPSL (turquoise) based segmentation of GTV. (b) Manual contour (red) compared with DIR based contour (turquoise).

**Figure 5:**
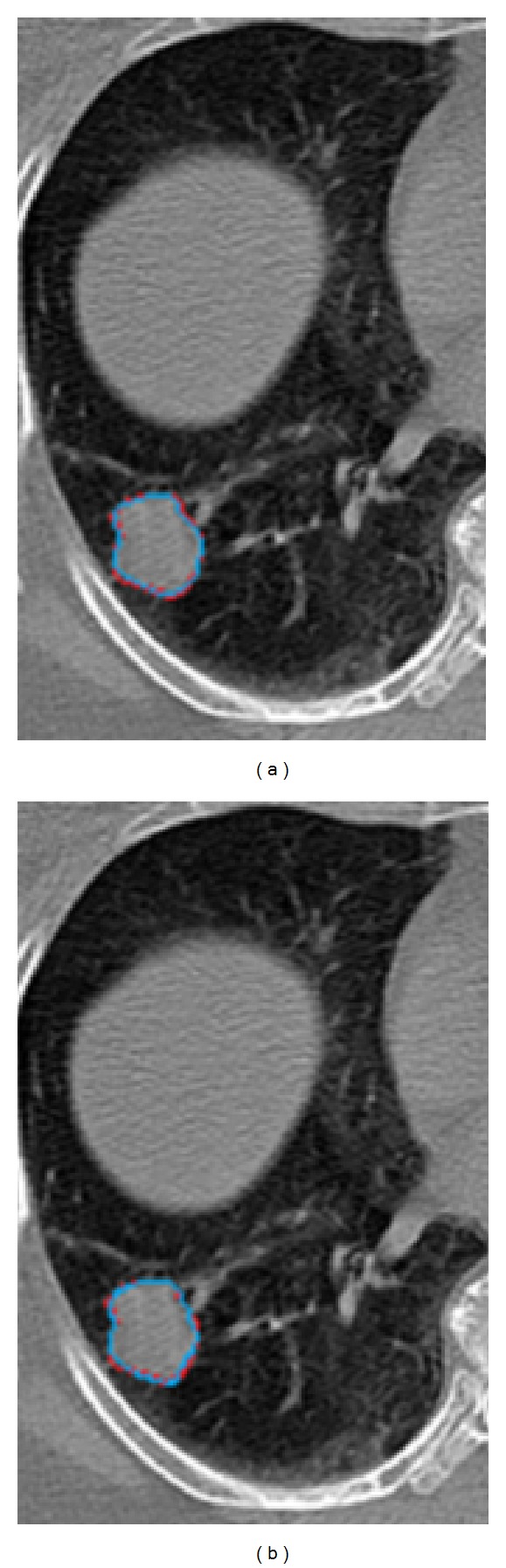
Comparison of automated contouring methods with ground-truth in case 3. (a) Manual contour (red) compared with MPSL (turquoise) based segmentation of GTV. (b) Manual contour (red) compared with DIR based contour (turquoise). Notice that diaphragm with similar intensity as that of the GTV was not included in the contour by the MPSL algorithm.

Figures 3, 4, and 5 shows the GTV segmentation using MPSL algorithm and DIR based segmentation for three representative cases.

Segmentation methods based on thresholding and region-growing would fail in the case shown in Figure 3 due to large connection between GTV and the rest of the body.

### 3.2. Time Performance Comparison

The time for the automatic segmentation of the GTV using MPSL in the 9 cases considered in this study was 24.2 ± 6.1 seconds per phase. In the case of DIR based segmentation of GTV, the manual contouring on the first phase using ITK-SNAP took 153.4 ± 153.2 seconds (note that the large standard deviation in the time for manual contouring was due to one outlier case which had GTV spanning over many slices and it took 538 seconds to manually contour the GTV). The DIR based segmentation using Elastix© software took 142.3 ± 11.3 seconds to calculate the deformation map and then transform the contour from the first phase to one another phase.

### 3.3. Motion Quantification

**Figure 6:**
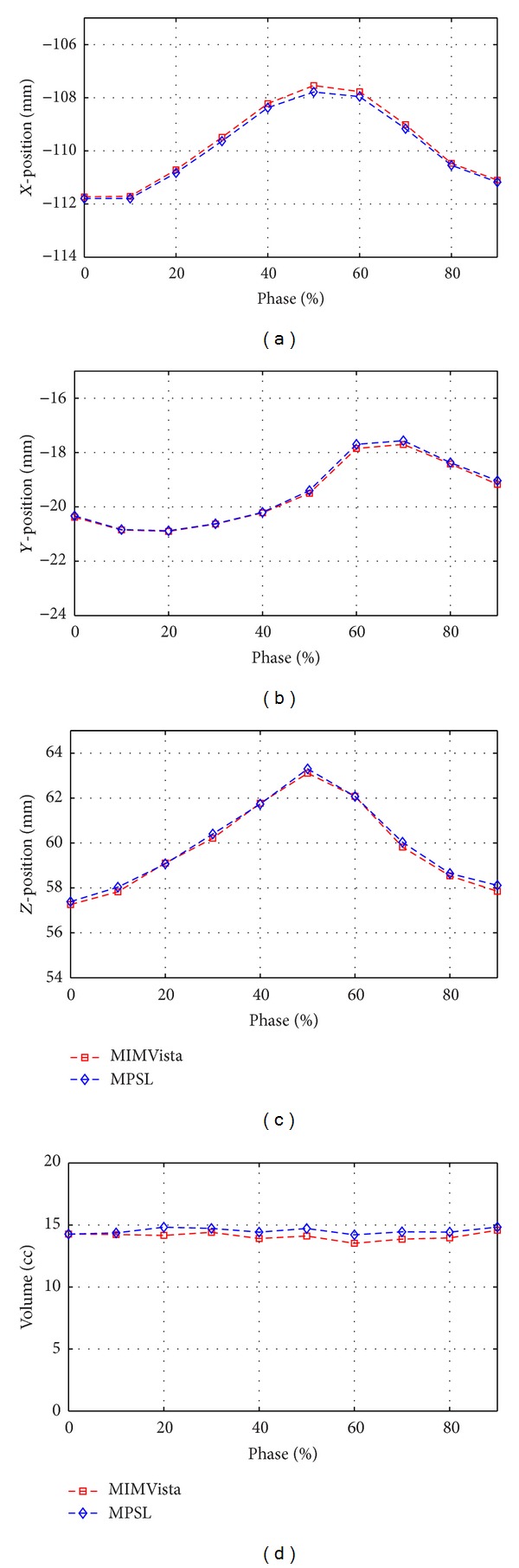
Good agreement of MPSL based estimation of GTV position and volume quantification with MIMVista based estimation for different phases of the 4DCT scan shown in a patient with 15cc GTV.

**Figure 7:**
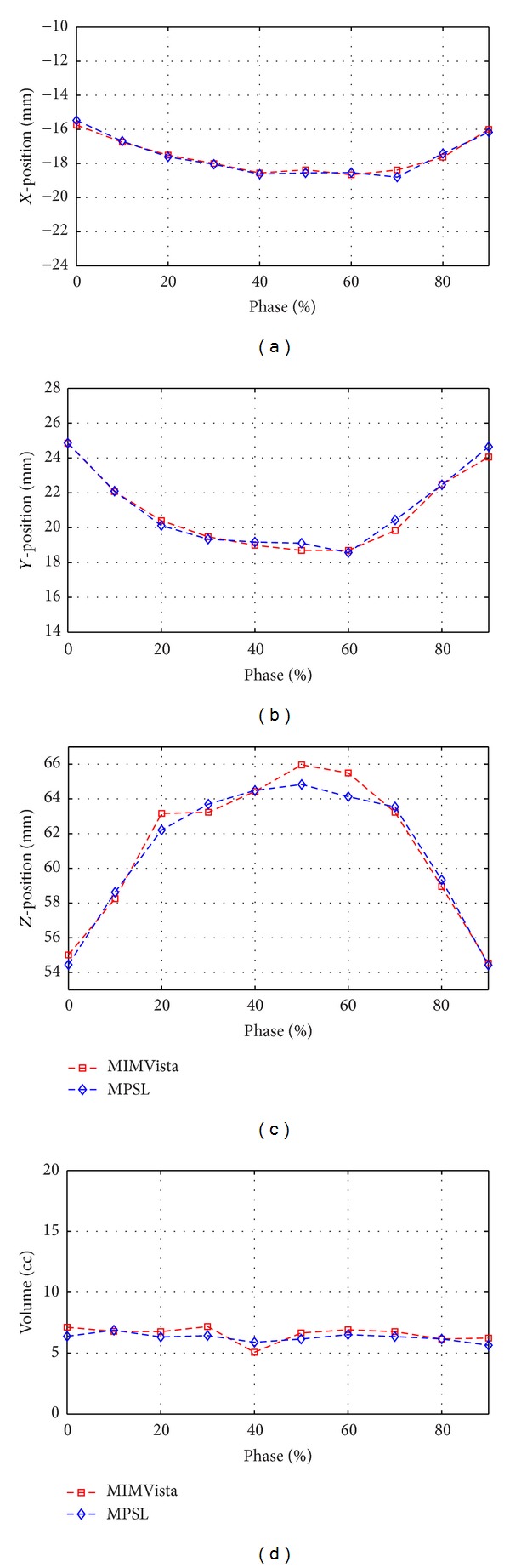
Good agreement of MPSL based estimation of GTV position and volume quantification with MIMVista based estimation for different phases of the 4DCT scan shown in a patient with 6cc GTV.

Figures 6 and 7 show the quantification of GTV motion in *x*, *y*, and *z* directions using MPSL for two representative cases. The MPSL based motion estimation was compared with motion estimation performed using ground-truth. In the result shown Figure 6 the ground-truth in all the different phase volumes was manually contoured, whereas, in Figure 7, the tumor in the volume corresponding to 40% phase was manually contoured and the contours were propagated to other phases using deformable registration. The figures show that MPSL segmentation based tumor position and volume quantification results are strongly correlated with the quantification results derived using MIMVista.

## 4. Discussion

MPSL-based segmentation was developed for rapid and accurate contouring of the GTV in different phases of 4D CT data. High Dice similarity (0.865 ± 0.037) with ground-truth and processing time of less than half a minute show the potential of MPSL for improving workflow in radiotherapy planning. MPSL method also achieved high sensitivity (0.827 ± 0.059) and PPV (0.912 ± 0.064). The MPSL algorithm achieved rapid segmentation in time scales shorter than the state-of-the-art deformable image registration based segmentation methods. In the case of a single 512 × 512 image, MPSL segmentation was achieved in time scale of a tenth of a second. For a 3D volume with in-plane resolution of 512 × 512 and 80–100 slices segmentation was achieved in less than 30 seconds. Furthermore, MPSL does not require manual contouring on one phase for propagating to other phases, facilitating efficient use without need for previous segmentation. The method is also less subjective. Therefore, the algorithm could be used in scenarios where fast contouring of the GTV is needed.

The paired *t*-test showed statistically significant difference for Dice coefficients and sensitivity between DIR and MPSL based segmentation methods. However, both had high values for Dice and sensitivity. DIR based segmentation was statistically closer to ground-truth as compared to MPSL based segmentation, but with nearly sixfold increase in the processing time. Furthermore, the cases with the greatest difference in the Dice similarity coefficient between MPSL and DIR based methods were the cases where a large region of the GTV was attached to the wall of the lung. In such cases, delineation of GTV in the “ground-truth” tends to be subjective. Hence, if the manual GTV contours on first phase are registered using deformable registration, higher agreement with ground-truth is expected as compared to MPSL because of the subjectivity propagated to the other phases. In those cases, the automatic segmentation algorithm makes morphological conclusion as to where the boundary will be, where the manual segmentation is bound to be subjective. In our data there were two such cases out of the nine. Omitting those two cases in the paired *t*-test analysis showed no statistical difference (*P* value of 0.09) between the Dice similarity coefficients from MPSL and DIR based methods.

In this work we have not used user-input to find the location of the GTV, which is attractive for ease and reproducibility. However, the algorithm could be modified to use input information regarding the location of GTV to further improve the speed and accuracy. Hence, a tool has been implemented in the software for the user to provide a “click” inside the tumor as a starting point, which allows the algorithm the parallel ability to use the local neighborhood intensity values as a range for thresholding. Similarly, if preexisting GTV contours on earlier scans do exist, a rigid registration, which is much faster than DIR, could be applied to get input information about the target location.

The computation complexity of MPSL was compared with DIR based segmentation algorithms available in Elastix© and MIMVista software. There are other nonrigid registration algorithms such as diffeomorphic symmetric normalization (DSN). Comparison of DSN methods is beyond the scope of this work. Parallel processing and multithreaded approaches could be used to reduce the computational tim But we have not explored those approaches. The algorithms developed in this work are applicable to MR data and CBCT in addition to 4DCT data used in this work. Future work would explore the use of MPSL approach for real-time segmentation of GTV on MR and CBCT data.

## 5. Conclusion

Automated and rapid generation of GTV contours using the MPSL algorithm may be advantageous for a number of scenarios including adaptive radiotherapy planning. The MPSL method achieved similar segmentation accuracy as compared to state-of-the-art deformable registration based segmentation methods, but with significant reduction in the computation time.

## Figures and Tables

**Table 1:** Dice similarity coefficients, sensitivity, and PPV between GTV contours generated with the proposed MPSL method and DIR based segmentation.

Case number	Dice			Sensitivity			PPV		
	MPSL versus manual	DIR versus manual	MPSL versus DIR	MPSL versus manual	DIR versus manual	MPSL versus DIR	MPSL versus manual	DIR versus manual	MPSL versus DIR
1	0.882	0.984	0.885	0.836	0.979	0.843	0.933	0.988	0.931
2	0.872	0.914	0.856	0.805	0.907	0.796	0.950	0.921	0.925
3	0.860	0.915	0.877	0.801	0.878	0.850	0.929	0.955	0.906
4	0.941	0.941	0.914	0.901	0.956	0.863	0.984	0.927	0.971
5	0.825	0.949	0.823	0.723	0.963	0.711	0.962	0.935	0.975
6	0.845	0.841	0.792	0.876	0.945	0.737	0.816	0.758	0.855
7	0.854	0.929	0.830	0.775	0.926	0.755	0.951	0.931	0.921
8	0.887	0.885	0.828	0.899	0.954	0.781	0.875	0.825	0.880
9	0.819	0.826	0.785	0.830	0.932	0.713	0.809	0.742	0.873

Average	0.865	0.909	0.843	0.827	0.938	0.783	0.912	0.887	0.915
St. dev.	0.037	0.051	0.043	0.059	0.031	0.059	0.064	0.089	0.042
